# Autocrine WNT signaling contributes to breast cancer cell proliferation via the canonical WNT pathway and EGFR transactivation

**DOI:** 10.1186/bcr1769

**Published:** 2007-09-26

**Authors:** Thomas Schlange, Yutaka Matsuda, Susanne Lienhard, Alexandre Huber, Nancy E Hynes

**Affiliations:** 1 Friedrich Miescher Institute for Biomedical Research, Maulbeerstrasse 66, CH-4058 Basel, Switzerland; 2 Université de Genève, Département de biologie moléculaire, Sciences II, 30 quai Ernest-Ansermet, CH-1211 Genève 4, Switzerland

## Abstract

**Background:**

De-regulation of the wingless and integration site growth factor (WNT) signaling pathway via mutations in APC and Axin, proteins that target β-catenin for destruction, have been linked to various types of human cancer. These genetic alterations rarely, if ever, are observed in breast tumors. However, various lines of evidence suggest that WNT signaling may also be de-regulated in breast cancer. Most breast tumors show hypermethylation of the promoter region of secreted Frizzled-related protein 1 (sFRP1), a negative WNT pathway regulator, leading to downregulation of its expression. As a consequence, WNT signaling is enhanced and may contribute to proliferation of human breast tumor cells. We previously demonstrated that, in addition to the canonical WNT/β-catenin pathway, WNT signaling activates the extracellular signal-regulated kinase 1/2 (ERK1/2) pathway in mouse mammary epithelial cells via epidermal growth factor receptor (EGFR) transactivation.

**Methods:**

Using the WNT modulator sFRP1 and short interfering RNA-mediated Dishevelled (DVL) knockdown, we interfered with autocrine WNT signaling at the ligand-receptor level. The impact on proliferation was measured by cell counting, YOPRO, and the MTT (3-[4,5-dimethylthiazol-2-yl]-2,5-diphenyl-tetrazolium bromide) assay; β-catenin, EGFR, ERK1/2 activation, and PARP (poly [ADP-ribose]polymerase) cleavages were assessed by Western blotting after treatment of human breast cancer cell lines with conditioned media, purified proteins, small-molecule inhibitors, or blocking antibodies.

**Results:**

Phospho-DVL and stabilized β-catenin are present in many breast tumor cell lines, indicating autocrine WNT signaling activity. Interfering with this loop decreases active β-catenin levels, lowers ERK1/2 activity, blocks proliferation, and induces apoptosis in MDA-MB-231, BT474, SkBr3, JIMT-1, and MCF-7 cells. The effects of WNT signaling are mediated partly by EGFR transactivation in human breast cancer cells in a metalloprotease- and Src-dependent manner. Furthermore, Wnt1 rescues estrogen receptor-positive (ER^+^) breast cancer cells from the anti-proliferative effects of 4-hydroxytamoxifen (4-HT) and this activity can be blocked by an EGFR tyrosine kinase inhibitor.

**Conclusion:**

Our data show that interference with autocrine WNT signaling in human breast cancer reduces proliferation and survival of human breast cancer cells and rescues ER^+ ^tumor cells from 4-HT by activation of the canonical WNT pathway and EGFR transactivation. These findings suggest that interference with WNT signaling at the ligand-receptor level in combination with other targeted therapies may improve the efficiency of breast cancer treatments.

## Introduction

Growth factors of the wingless and integration site growth factor (WNT) family are secreted, glycosylated, and palmitoylated peptides that interact with seven-transmembrane receptors of the Frizzled (FZD) family. Diverse signaling pathways are activated upon WNT/FZD binding. The ligand/receptor interaction has been shown to induce the phosphorylation of scaffolding proteins of the Dishevelled (DVL) family by casein kinase Iε and -2 and PKCα [1-3]. This event was reported to be a component of all WNT-induced signaling pathways [4,5]. The so-called canonical WNT signaling pathway leads to stabilization of β-catenin through inactivation of a protein complex consisting of, amongst others, the tumor suppressors APC and Axin. This destruction complex normally triggers rapid β-catenin phosphorylation, inducing its ubiquitination and degradation. In the presence of canonical WNT ligands, β-catenin is stabilized, binds transcription factors of the LEF-1/T-cell factor (TCF) family, and stimulates target gene transcription [6].

Aberrant activation of the WNT signaling pathway plays an important role in the development of many human cancer types. In colorectal cancer (CRC), mutations in APC, axin, or β-catenin itself promote β-catenin stabilization and transcription of target genes encoding cancer-associated proteins [7]. In contrast to CRC, WNT pathway mutations rarely, if ever, are detected in breast tumors [8]. However, various lines of evidence suggest that, in breast cancer, the WNT pathway may be de-regulated by loss of expression of negative pathway regulators. For example, expression of the extracellular inhibitor of WNT signaling, secreted Frizzled-related protein 1 (sFRP1), which competes with FZD receptors for ligand binding, is downregulated in many breast tumors and is associated with poor prognosis [9-11]. Furthermore, many studies have reported that WNT ligands and FZD receptors are expressed in human breast cancer cell lines and primary tumors [7,12-14]. Finally, β-catenin is frequently found stabilized and nuclear in human breast tumors and this finding has been associated with poor prognosis [15]. Taken together, these observations suggest that WNT signaling may frequently be de-regulated in breast cancer.

We have previously described a novel crosstalk between WNT signaling and epidermal growth factor receptor (EGFR) [16]. The mechanism, which we have shown to involve activation of zinc-dependent membrane-associated metalloproteases [16] that control the cleavage and availability of ERBB ligands [17], appears to be analogous to that described for transactivation of EGFR triggered by stimulation of G protein-coupled receptors (GPCRs) [18]. GPCR-mediated EGFR transactivation involves various heterotrimeric G protein α subunits, activation of PKC and/or Src kinase, as well as ADAMs (A Disintegrin And Metalloprotease) (reviewed recently in)[19]) or matrix metalloprotases (MMPs) [20].

In this study, we provide evidence for constitutive autocrine WNT signaling in human breast cancer cells. We show that sFRP1 blocks proliferation of many breast tumor cell lines through interference with pathway activation that is presumably driven by endogenous WNT ligands. Thus, our study clearly demonstrates that sFRP1 fulfills its proposed tumor suppressor function [21]. Downstream of the WNT ligand/FZD receptor interaction, knockdown of DVL expression using short interfering RNA (siRNA) also results in a proliferative reduction and the induction of apoptosis in many human breast cancer cell lines. Our results, showing that Wnt1 transactivates EGFR in tumor cell lines, imply that, in breast cancer, constitutive WNT signaling might impact not only on the canonical pathway, but also on EGFR activity by stimulating ligand availability. Considering that constitutive ERBB receptor activation is an important mechanism promoting cancer cell proliferation, migration [22,23], and sensitivity to anti-cancer therapies [24], approaches to target WNT pathway activity might be appropriate as an anti-cancer strategy.

## Materials and methods

### Reagents

The following antibodies were used in this study: extracellular signal-regulated kinase 1/2 (ERK1/2), p-ERK1/2, total β-catenin, poly(ADP-ribose)polymerase (PARP), EGFR, p-EGFR (tyrosine [Tyr] 845), and p-Tyr-100 (Cell Signaling Technology, Inc., Danvers, MA, USA); c-MYC (9E10), DVL2 and DVL3, EGFR 528, 1005, and R1 (Santa Cruz Biotechnology, Inc., Santa Cruz, CA, USA); Wnt1 and DVL1 (R&D Systems Europe, Abingdon, UK); active β-catenin (anti-ABC; Upstate, now part of Millipore Corporation, Billerica, MA, USA); and α-Tubulin (Lab Vision Corporation, Fremont, CA, USA). As secondary antibodies, α-rabbit and α-mouse (GE Healthcare, Little Chalfont, Buckinghamshire, UK, and LI-COR Biosciences, Lincoln, NE, USA) or α-goat (DAKO A/S, Glostrup, Denmark) coupled to horseradish peroxidase (HRP) were used. Mouse Wnt1 in the retroviral vector pLNCX was obtained from Andrew McMahon (Harvard University, Cambridge, MA, USA); the cDNA encoding human sFRP1 in pcDNA was provided by Jeffrey Rubin (National Cancer Institute, Bethesda, MD, USA). The retroviral vector for the expression of short hairpin RNA (shRNA) constructs pSUPERretro Neo green fluorescent protein (GFP) was provided by Francois Lehembre (DKBW, Basel, Switzerland). PKI166 and AEE788 were provided by Peter Traxler (Novartis Pharma AG, Basel, Switzerland); CGP77675 was provided by Jonathan Green and Mira Susa Spring (Novartis Pharma AG), and CGS27023A was provided by Ulf Neumann (Novartis Pharma AG). 4-Hydroxytamoxifen (4-HT) was purchased from Sigma-Aldrich (St. Louis, MO, USA).

### Cell culture, transfections, and retroviral infections

The human breast cancer cell lines T47D, MCF-7, ZR-75.1, SkBr3, BT474, and MDA-MB-231 (American Type Culture Collection, Manassas, VA, USA) and JIMT-1 (DSZM, Braunschweig, Germany) were cultivated in Dulbecco's modified Eagle's medium (DMEM), 10% heat-inactivated fetal calf serum (FCS) (Amimed, Allschwil, Switzerland) supplemented with penicillin and streptomycin. HC11 and HC11/Wnt1 cells were maintained in RPMI 1640, 10% FCS, penicillin/streptomycin, epidermal growth factor (EGF) (Collaborative Research Co., Bedford, MA, USA) and insulin (Sigma-Aldrich). HC11/Wnt1 cells were kept under selection in 1 mg/mL G-418 (Life Technologies, Inc., now part of Invitrogen Corporation, Carlsbad, CA, USA). HEK 293 cells were transfected with a vector encoding myc/HIS-tagged human sFRP1 using Lipofectamine according to the manufacturer's guidelines. Cells were kept for 3 weeks in medium containing 1.5 mg/mL G-418, and clones were selected. T47D and SkBr3 cells were stably transfected with Wnt1 or empty pLNCX as control by Lipofectamine Reagent (Invitrogen Corporation) according to the manufacturer's instructions. Clones of Wnt1-expressing cells were selected with 0.5 mg/mL G-418. The expression of Wnt1 ligand was verified by Western blotting, and biological activity was assayed in a co-culture assay with HEK 293/8× SUPERTopFlash cells, using 300,000 cells each in a six-well overnight culture before the assay was performed. Knockdown of β-catenin was achieved by retroviral infection [25] with pSUPERretro Neo GFP containing a short-hairpin targeting β-catenin [26]. A construct targeting bacterial LacZ (sense strand gCggCTgCCggAATTTACCdTdT) was used as control. Clones and a pool of cells with low levels of β-catenin were analyzed for their response to Wnt1 condition medium (CM). Src^-/- ^mouse embryonic fibroblasts (MEFs), provided by Kurt Ballmer (Paul Scherrer Institut, Villigen, Switzerland), were transfected with empty vector or a c-Src-expressing vector, and clones were selected. Src re-expressing MEFs were generated by Monilola Olayioye (University of Stuttgart, Germany).

### siRNA transfections

Five hundred thousand cells per well were seeded in a six-well plate the day before transfection and were transfected with either 50 nM control RNA duplex targeting bacterial LacZ (sense strand gCggCUgCCggAAUUUACCdTdT) or a mixture of two siRNA duplexes (25 nM each; Qiagen GmbH, Hilden, Germany) targeting bases 1420 to 1440 (gCUCAACAAgAUCACCUUCUdTdT) in human DVL1 (NM_004421) and bases 1754 to 1774 and 1579 to 1599 (gUCAACAAgAUCACCUUCUdTdT) in human DVL2 (NM_004422) and DVL3 (NM_004423), respectively, using HiPerfect (Qiagen GmbH) according to the manufacturer's instructions. The DVL target sequences were chosen based on the high conservation in all three human DVL homologues. The cells were cultured for 72 hours, and knockdown efficiency was monitored by Western blotting.

### Conditioned media

Confluent HC11/Wnt1 or parental HC11 cells were cultured for 3 days in RPMI 1640, 10% FCS, penicillin/streptomycin without EGF, insulin, and G-418. The supernatant was filtered through a 0.25-μm syringe filter (Sarstedt AG & Co., Nümbrecht, Germany). Biological activity of Wnt1 CM and control CM was assayed by their ability to induce β-catenin/TCF-dependent luciferase reporter activity in HEK 293/8× SUPERTopFlash cells (provided by Feng Cong, Novartis Institutes for BioMedical Research, Cambridge, MA, USA).

sFRP1 CM was obtained from HEK 293 cells transfected with myc/HIS-tagged human sFRP1 cDNA. CM was collected and sFRP1 activity was assayed by testing its ability to block the activation of β-catenin/TCF-driven transcription in a co-culture of T47D/Wnt1 cells and HEK 293/8× SUPERTopFlash cells and the reduction of DVL3 phosphorylation in T47D/Wnt1 cells. For treatment of breast cancer cell lines, confluent sFRP1-expressing HEK 293 cells were treated overnight with 10 mM sodium butyrate in 0.1% FCS to increase sFRP1 expression. The CM was concentrated, and sodium butyrate was removed by filtration with a Centricon Plus-70 filtration unit (Millipore Corporation). The resulting concentrate was diluted to the starting volume or used as a 2× concentrate and adjusted to 10% FCS accordingly. Cell proliferation was measured either by counting cell numbers manually or with a Vi-Cell XR cell viability analyzer (Beckman Coulter, Nyon, Switzerland), Cell Proliferation Kit I (MTT; Roche Diagnostics GmbH, Mannheim, Germany), or YOPRO cell viability assay (Invitrogen Corporation) according to manufacturer instructions.

Hybridoma cells secreting the EGFR monoclonal antibody C225 were cultured in DMEM, 10% FCS. Collected medium was cleared by centrifugation, filtered, and used undiluted on target cells for 2 hours prior to collection of cell lysates.

### Purification of sFRP1

sFRP1 was purified by fast performance liquid chromatography from sFRP1 CM. After 1:10 dilution in 50 mM sodium phosphate loading buffer pH 7.0, the solution was loaded on a 1 mL HiTrap-HIS column (GE Healthcare) that was previously loaded with 1 mL 0.5 M NiSO_4 _and washed with 10 column volumes of loading buffer. Elution was performed using 50 mM sodium phosphate, 100 mM NaCl pH 7.0 elution buffer with a 3-minute step-gradient of 10 to 500 mM imidazole. Fractions were collected, and 1-μL aliquots were analyzed by Western blotting using a c-MYC antibody for detection of the MYC-tag. Biological activity was assayed as previously described for sFRP1 CM, and the identity of the purified protein was determined by mass spectrometry.

### Protein extraction, immunoprecipitation, and Western blotting

Cells were lysed in lysis buffer (1% Nonident P-40, 50 mM Tris pH 7.5, 120 mM NaCl, 5 mM EDTA [ethylenediaminetetraacetic acid], 1 mM EGTA [ethylene glycol tetraacetic acid], 2 mM sodium vanadate, 20 mM β-glycerophosphate, 10 μg/mL aprotinin, 10 μg/mL leupeptin, 0.5 mM PMSF [phenylmethylsulphonyl fluoride], 50 mM NaF, and 1 mM dithiothreitol) for 5 minutes on ice, and lysates were collected. For a Western analysis, loading buffer was added to 30 to 50 μg of protein and the samples were denatured for 10 minutes at 95°C prior to separation on 10% polyacrylamide gels and blotting by semi-dry transfer for 90 minutes on polyvinylidene fluoride membrane (Millipore Corporation). Blots were pre-blocked using 10% horse serum in TBS-T buffer for 1 hour (0.2 M NaCl, 25 mM Tris pH 7.5, 0.5 mL/L Tween-20) and incubated with primary antibodies at room temperature for 1 hour or at 4°C overnight, followed by 30 minutes of incubation with secondary antibodies: α-rabbit-HRP, α-mouse-HRP, or α-goat-HRP (1:5,000). Detection of luminescence was carried out using ECL (enhanced chemiluminescence) (GE Healthcare) or SuperSignal West Dura (Pierce, Rockford, IL, USA) according to manufacturer instructions. Immunoprecipitations (IPs) and Western analyses were performed using standard procedures [24,27]. EGFR IP was performed with α-EGFR 528 and R1. Quantifications of Western blots were carried out using the ImageQuant TL version 2005 software package from Amersham Biosciences (now part of GE Healthcare).

## Results

### WNT pathway activity in human breast tumor cell lines

WNTs activate multiple intracellular signaling cascades, including the canonical pathway that promotes β-catenin stabilization and TCF-mediated transcription [6] and other non-canonical pathways, one being Wnt-mediated EGFR transactivation [16]. To explore the possibility that Wnt signaling is de-regulated in breast cancer by autocrine pathway activation, we examined breast cancer cell lines for signs of canonical pathway activity and for crosstalk between WNT, EGFR, and ERK1/2 signalling. The panel includes the luminal, estrogen receptor-positive (ER^+^) T47D, MCF-7, and ZR75.1 cells, the ERBB2-overexpressing SkBr3, JIMT-1, and BT474 cells, and the basal B, ER-negative MDA-MB-231 cells [28].

**Figure 1:**
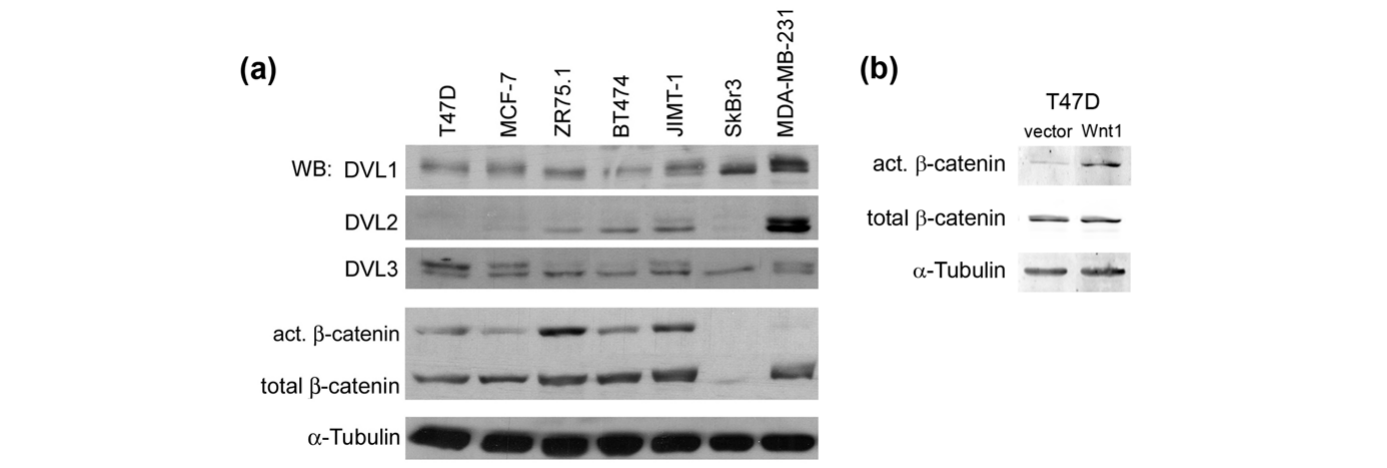
Autocrine WNT signaling in breast cancer cell lines. **(a) **Lysates from the indicated human breast cancer cell lines were analyzed by SDS-PAGE followed by immunoblotting for Dishevelled 1 (DVL1), DVL2, and DVL3 (the upper band indicates the phosphorylated form of each), active β-catenin, total β-catenin, and α-Tubulin as a loading control. **(b) **Lysates from T47D/Wnt1 cells and vector control were analyzed by SDS-PAGE/immunoblotting for active β-catenin, total β-catenin, and α-Tubulin. WB, Western blotting; WNT, wingless and integration site growth factor.

As a consequence of WNT binding to FZD, cytoplasmic scaffolding proteins of the Dishevelled family (DVL1, DVL2, and DVL3) become phosphorylated on serine and threonine residues. DVL phosphorylation is the most proximal signaling event downstream of the WNT-mediated activation of FZD and can be monitored by a decrease in the electrophoretic mobility of p-DVL [4]. To date, DVL phosphorylation has been shown to be mediated only by WNT signaling and DVL is upstream of all known WNT-induced signaling pathways. DVL1 and DVL3 were consistently expressed at relatively uniform levels in all the breast cancer cell lines, whereas DVL2 was expressed in a more differential manner (Figure 1a). Bands corresponding to p-DVL1 and/or p-DVL3 were detected in all of the cell lines. p-DVL2 was also high in MDA-MB-231 cells (Figure 1a). These results suggest that WNT signaling might be activated in an autocrine fashion in each of the examined breast cancer cell lines.

As a read-out for activation of the canonical WNT pathway, active, unphosphorylated β-catenin (act. β-catenin) was analyzed in these breast cancer cell lines and in a control T47D cell line engineered to ectopically express Wnt1 (Figure 1b). Control and T47D/Wnt1 cells have the same level of total β-catenin. Importantly, the Wnt1-expressing T47D cells have an approximately three-fold increase in active β-catenin levels compared with control cells (Figure 1b), attesting to the ability of the antiserum to measure canonical pathway activity. In the majority of the breast tumor cell lines, active β-catenin was present at various levels (Figure 1a). Only in SkBr3 cells, which have very low total β-catenin levels, no active β-catenin protein was detected. These results imply that the canonical WNT signaling pathway is constitutively active in most breast tumor cell lines.

### *In vitro *effects of sFRP1 on proliferation of human breast cancer cell lines, canonical β-catenin signaling, and ERK activity

**Figure 2:**
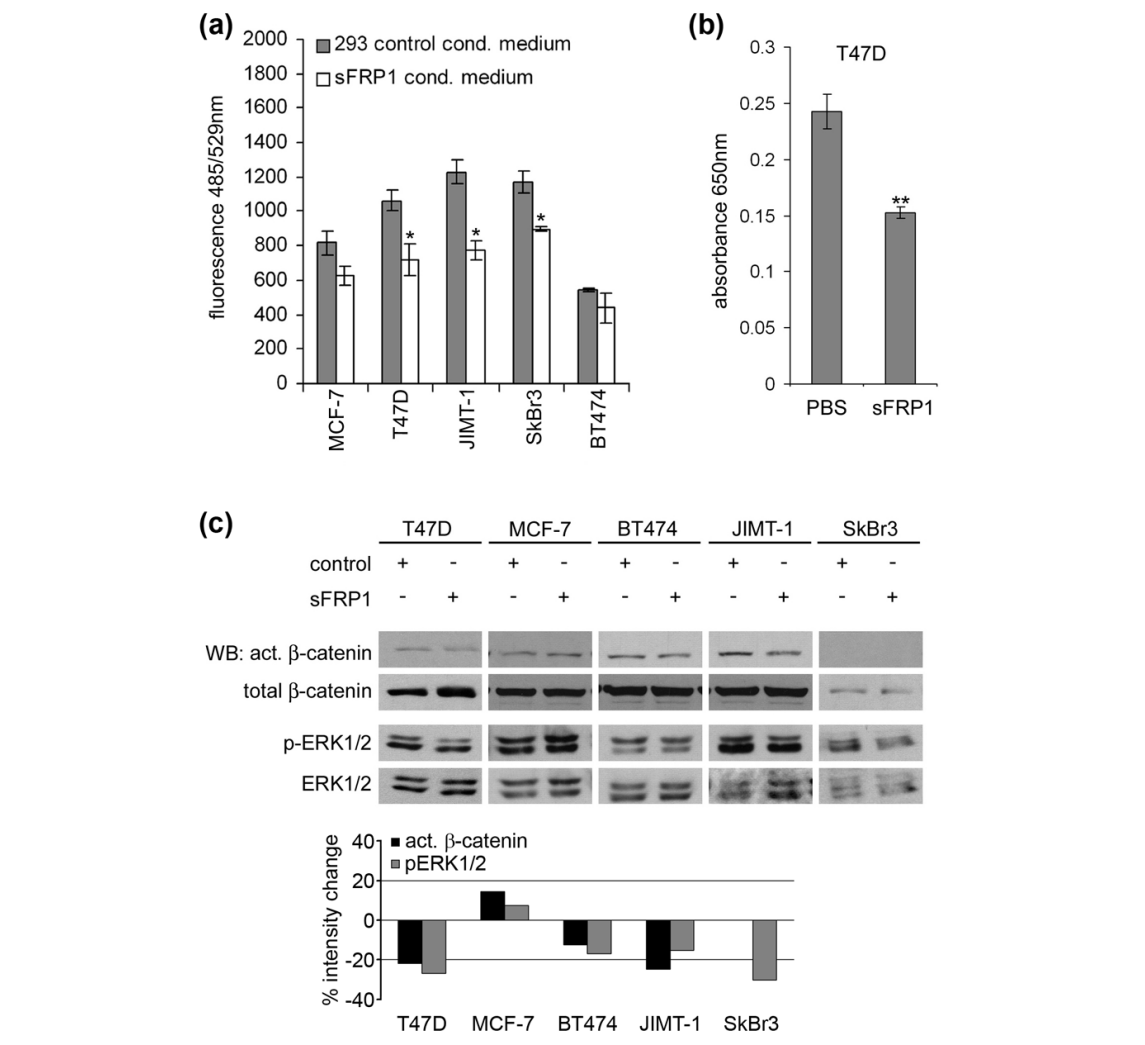
Treatment of human breast cancer cells with secreted Frizzled-related protein 1 (sFRP1) reduces proliferation and impairs canonical wingless and integration site growth factor signaling and extracellular signal-regulated kinase 1/2 (ERK1/2) phosphorylation. **(a) **One thousand to 5,000 cells of the indicated cell lines were seeded in a 96-well plate, and proliferation was measured in a YOPRO assay after 3 days of treatment with sFRP1 conditioned medium (CM) or control CM. **(b) **T47D cells were treated for 3 days with 30 μg/mL purified sFRP1 or phosphate-buffered saline, and cell numbers were measured in an MTT (3-[4,5-dimethylthiazol-2-yl]-2,5-diphenyl-tetrazolium bromide) assay. The results represent the mean of three experiments (± standard error). *p < 0.05, **p < 0.005, unpaired Student *t *test, comparison to corresponding control-treated cell line. **(c) **The indicated human breast cancer cell lines were treated for 2 hours with concentrated sFRP1 CM, and cell lysates were analyzed by SDS-PAGE/immunoblotting for active β-catenin, total β-catenin, p-ERK1/2, and ERK1/2 (upper panel). The results were quantified using ImageQuant (lower panel).

Since sFRP1 expression is lost in primary breast tumors and tumor cell lines by promoter hypermethylation [10,29], this might be one mechanism contributing to WNT pathway activity. We therefore assessed the effect of blocking WNT pathway activity on *in vitro *proliferation of breast tumor cell lines. Treatment of T47D cells with either purified sFRP1 or sFRP1 CM blocked their proliferation by 30% (Figure 2a,b). Proliferation of JIMT-1, SkBr3, and MDA-MB-231 cells was also significantly inhibited (20% to 30%) by sFRP1 CM, whereas BT474 and MCF-7 cells were not significantly affected by the treatment (Figure 2a).

To analyze the signaling pathways involved in the anti-proliferative activity of sFRP1, we examined its effects on canonical WNT signaling, which, as shown above (Figure 1a), is constitutively active in most of the examined breast tumor cell lines. Treatment of T47D, BT474, and JIMT-1 cells with sFRP1 CM caused a 10% to 20% reduction in active β-catenin levels, whereas there was no observable decrease in MCF-7 cells (Figure 2c, quantified below). These results suggest that, in these three cell lines, β-catenin stabilization is at least partly due to autocrine activation of the pathway by WNT ligands that can be blocked from binding their cognate FZD receptor by sFRP1.

As we have previously shown that Wnt growth factors activate the ERK1/2 pathway in mouse mammary epithelial cells [16], we next examined the effect of sFRP1 on ERK1/2 activity. sFRP1 treatment lowered the basal level of p-ERK1/2 in all cell lines analyzed with the exception of MCF-7 (Figure 2c), which also showed no decrease in active β-catenin in response to sFRP1. These results are in good agreement with those showing that sFRP1 treatment reduced proliferation of T47D, JIMT-1, and SkBr3 cells, but not of MCF-7 cells. In summary, these results show that, in some breast cancer cell lines, both canonical and non-canonical Wnt signaling can be blocked by sFRP1 treatment. Furthermore, they suggest that sFRP1 has the potential to act as an anti-proliferative agent.

### siRNA-mediated knockdown of DVL reduces c-MYC expression and induces apoptosis

**Figure 3:**
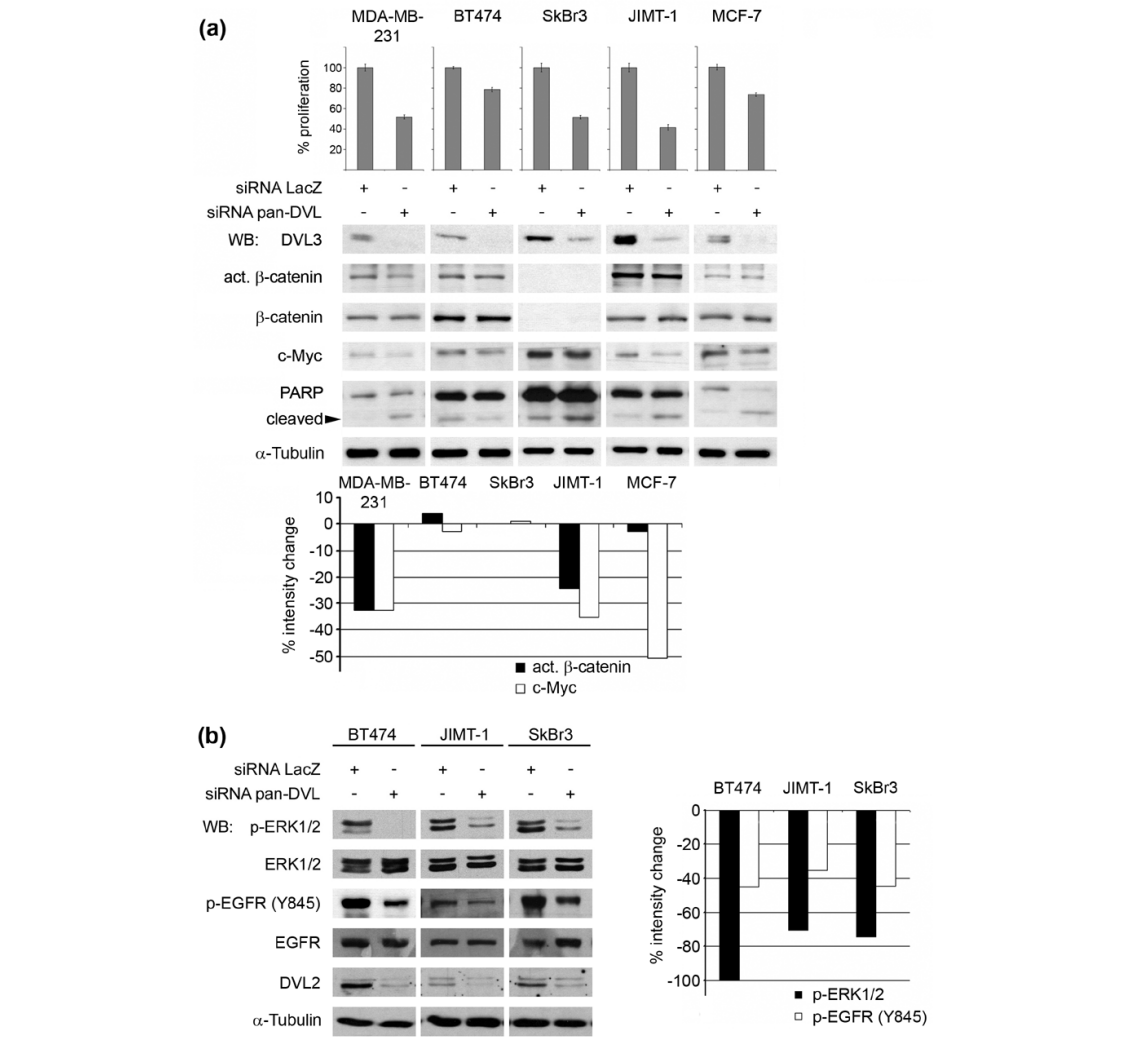
Short interfering RNA (siRNA)-mediated knockdown of Dishevelled (DVL) homologues results in decreased canonical wingless and integration site growth factor (WNT) signaling, a reduction in basal epidermal growth factor receptor (EGFR) and extracellular signal-regulated kinase 1/2 (ERK1/2) activation, and the induction of apoptosis in human breast cancer cells. **(a) **The indicated human breast cancer cell lines were transfected with pan-DVL siRNA. Two thousand to 5,000 cells were seeded in triplicate in 12-well plates the day after the transfection, and the cell number was counted after 7 days using a Vi-Cell XR cell viability analyzer. DVL knockdown was verified by SDS-PAGE/immunoblotting (only DVL3 is shown). The levels of act. β-catenin, total β-catenin, the WNT target c-MYC, and poly(ADP-ribose)polymerase (PARP) were analyzed by SDS-PAGE/immunoblotting. The lower band (80 kDa) in the blot probed for PARP represents the cleavage product upon induction of apoptosis. α-Tubulin was used as a loading control. For quantification, act. β-catenin levels were normalized to total β-catenin and c-MYC was normalized to α-Tubulin expression. **(b) **The indicated human breast cancer cell lines were transfected with pan-DVL siRNA and analyzed by SDS-PAGE/immunoblotting for p-ERK1/2 and EGFR Y845 phosphorylation. DVL2 levels are shown to monitor efficient knockdown of DVL, and α-Tubulin was used as loading control. For quantification, p-ERK1/2 was normalized to total ERK1/2 and p-EGFR Y845 was normalized to total EGFR expression.

**Figure 4:**
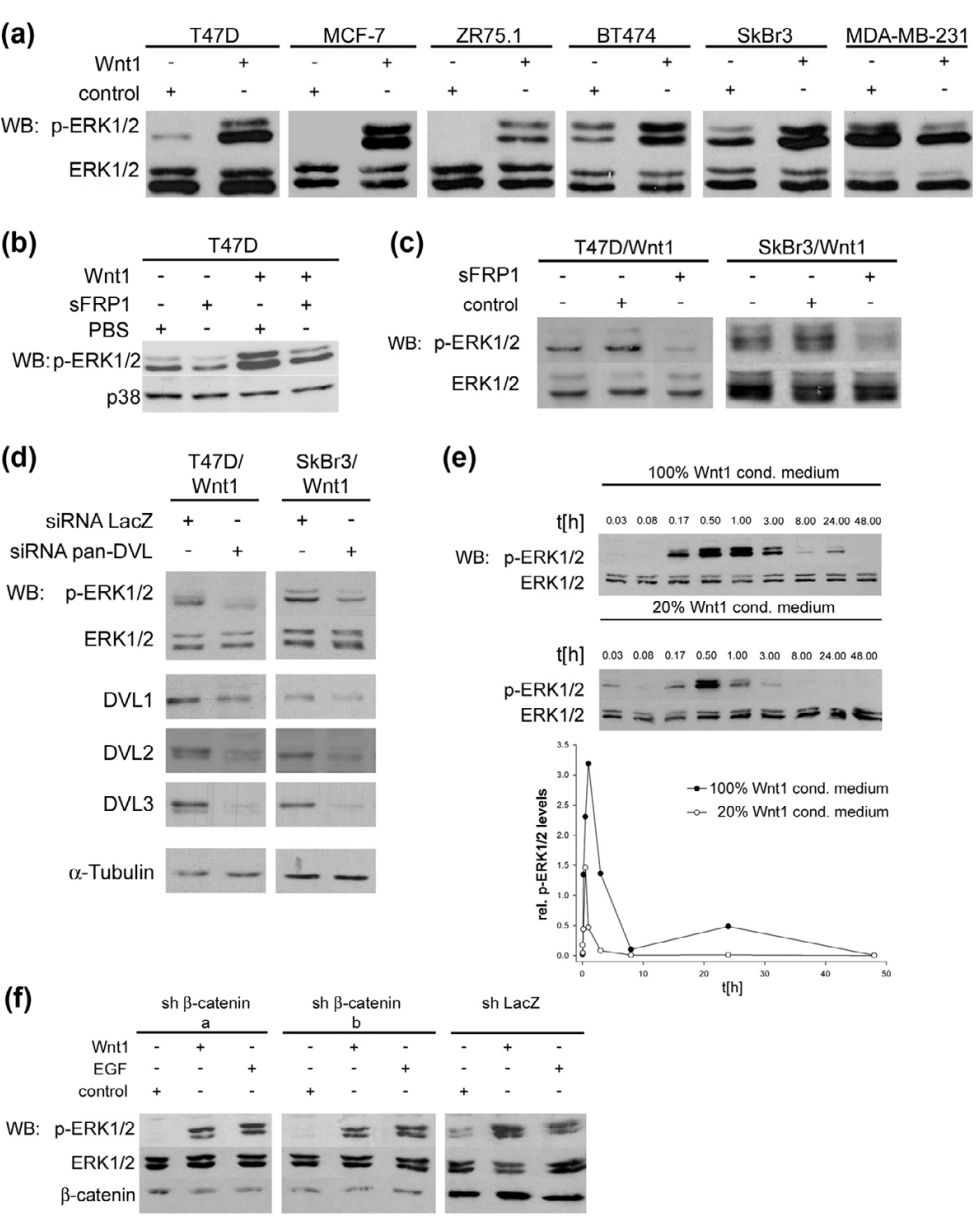
Wnt1 induces rapid phosphorylation of extracellular signal-regulated kinase 1/2 (ERK1/2) in human breast cancer cells. **(a) **Cultures of the indicated cell lines were treated for 20 minutes with Wnt1 conditioned medium (CM) or control CM, and lysates were analyzed by SDS-PAGE followed by immunoblotting for p-ERK1/2 and ERK1/2. **(b) **T47D cells were treated with control CM or Wnt1 CM, which was previously incubated for 2 hours with 30 μg/mL purified secreted Frizzled-related protein 1 (sFRP1) or phosphate-buffered saline as control. Cell lysates were analyzed by SDS-PAGE/immunoblotting for p-ERK1/2 and p38 as loading control. **(c) **Stably Wnt1-transfected T47D and SkBr3 cells were treated for 2 hours with sFRP1 CM, control CM, or normal growth medium. Total lysates were analyzed by SDS-PAGE followed by immunoblotting for p-ERK1/2 and ERK1/2. **(d) **T47D/Wnt1 and SkBr3/Wnt1 cells were seeded at 300,000 cells per well in a six-well plate the day before short interfering RNA (siRNA) transfection with either a LacZ control siRNA or pan-DVL siRNA. The cells were cultured for an additional 48 hours under normal growth conditions and 24 hours in 0.1% fetal calf serum before harvesting. Total lysates were analyzed by SDS-PAGE/immunoblotting for p-ERK1/2, ERK1/2, DVL1, DVL2, and DVL3, or α-Tubulin as a loading control. **(e) **T47D cultures were treated for the indicated times with 100% and 20% vol/vol Wnt1 CM. Total lysates were analyzed by SDS-PAGE/immunoblotting for p-ERK1/2 and ERK1/2 (upper panel). ERK activation was quantified after normalization of signal intensity of p-ERK1/2 to total ERK1/2 using the ImageQuant software. ERK activity peaks at between 30 minutes and 1 hour (lower panel). **(f) **Wnt1-mediated effects are independent of β-catenin. T47D cells were infected with a retrovirus carrying an expression cassette for a short-hairpin RNA targeting β-catenin. Two independent shβ-catenin clones and a control pool infected with a short hairpin against bacterial LacZ were treated with control CM or Wnt1 CM for 20 minutes or 10 ng/mL EGF for 5 minutes. Total lysates were analyzed by SDS-PAGE/immunoblotting for p-ERK1/2, ERK1/2, and β-catenin. DVL, Dishevelled.

Human breast cancer cells express multiple WNT ligands and FZD receptors [7,12-14], and it is likely that different sFRP family members interfere with only a subset of ligands [30]. Therefore, we hypothesized that knockdown of DVL homologues would lead to a stronger blockade of autocrine WNT signaling. Introduction of two siRNAs that target the three human DVL homologues (pan-DVL siRNA) achieved a strong knockdown of DVL2 and DVL3 in the cells; DVL1 was decreased approximately 50% with this siRNA (Figure 4d). siRNA-mediated DVL knockdown blocked proliferation of human breast cancer cells by 20% to 60% 7 days after transfection as determined by cell counting after viability staining (Figure 3a), with the most prominent effect in JIMT-1, SkBr3, and MDA-MB-231 cells, whereas BT474 and MCF-7 cells are less affected. As expected, DVL knockdown affects canonical WNT signaling activity since the level of active β-catenin decreases concomitantly with a reduction in c-MYC, a canonical WNT target (Figure 3a). SkBr3 cells show no reduction in c-MYC levels upon DVL knockdown, very likely because either *c-MYC *is amplified or canonical signaling is impaired since there is no active β-catenin in these cells. Finally, we observe an increase in PARP cleavage after DVL knockdown in all cell lines analyzed (Figure 3a), indicating that apoptosis is induced in all but BT474 cells. These data show that autocrine WNT signaling is required for proliferation and survival of human breast cancer cells.

### Downregulation of DVL in breast cancer cells lowers EGFR and ERK activity

Multiple mechanisms contribute to the autocrine ligand-induced EGFR activity [31] that is detected in many human tumors [32,33]. Given our previous results on WNT-induced EGFR transactivation [16], we considered it possible that WNT signaling might also play a role in some breast tumors. Thus, we asked whether WNT signaling also contributes to EGFR activity, concentrating on three cell lines, BT474, JIMT-1, and SkBr3, that in addition to ERBB2 overexpression have high levels of active EGFR and p-ERK1/2 (Figure 4a) [24,34,35]. pan-DVL knockdown lowered EGFR activity, as shown by a decrease in pY845 levels, and strongly reduced ERK1/2 activity in each of these cancer cell lines (Figure 3b). In summary, the results suggest that, in the examined breast cancer cell lines, WNT activity contributes to autocrine EGFR activation and ERK1/2 activity.

### Wnt1 induces ERK1/2 activity independently of canonical WNT signaling

In light of these results, we asked whether WNT ligands induce EGFR/ERK1/2 activation in human breast cancer cells in a fashion similar to that in non-transformed mouse mammary epithelial cells [16]. Wnt1 is not commercially available in a bioactive form and our own efforts to purify the protein using published protocols have failed. Our approaches to prove the specificity of Wnt1 action on ERK1/2 activity relied on the use of CM in combination with the natural WNT inhibitor sFRP1 and on ectopic expression of Wnt1 in breast cancer cell lines. Furthermore, we knocked down expression of DVL, the central WNT signaling mediators downstream of WNT-ligand-triggered FZD activation.

Cells were treated for 20 minutes with Wnt1 CM or control CM, and p-ERK1/2 levels were examined (Figure 4a). The ER^+ ^tumor cells T47D, MCF-7, and ZR75.1 have low basal p-ERK1/2 levels that strongly increased in response to Wnt1 treatment. The ERBB2-overexpressing tumor cells BT474 and SkBr3 have high basal p-ERK1/2, and both showed a further increase in ERK1/2 activity in response to Wnt1. p-ERK1/2 levels were not stimulated by Wnt1 treatment of MDA-MB-231 tumor cells, which have a *K-RAS *mutation and high basal ERK1/2 activity (Figure 4a) [36]. Wnt1 CM effects on ERK1/2 activity were blocked in T47D cells simultaneously treated with sFRP1 (Figure 4b). Similarly, when T47D/Wnt1 or SkBr3/Wnt1 cells were treated with sFRP1 for 2 hours prior to lysis of the cells, the level of ERK1/2 phosphorylation was strongly decreased (Figure 4c). This strongly suggests that the response in ERK1/2 phosphorylation toward Wnt1 treatment or stable Wnt1 expression is Wnt ligand-specific. This finding is supported by interference with WNT signaling downstream of the FZD receptor level through DVL knockdown that abolishes the increase in ERK1/2 phosphorylation in T47D/Wnt1 and SkBr3/Wnt1 cells (Figure 4d).

To assess the involvement of canonical β-catenin-dependent WNT signaling in the activation of ERK1/2 pathway, we next examined the kinetics of Wnt1-induced ERK1/2 activation after treating T47D cells with concentrated and with five-fold diluted Wnt1 CM. In both cases, ERK1/2 activation was rapid, peaking at between 30 and 60 minutes and falling back to basal by 8 hours (Figure 4e). Whereas the p-ERK1/2 levels were lower in cells treated with diluted Wnt1 CM, the kinetics were identical (Figure 4e). The rapid nature of ERK1/2 phosphorylation in response to Wnt1 makes it unlikely that transcriptional activity driven by canonical WNT/β-catenin signaling contributes to transactivation. However, to directly exclude this, β-catenin was knocked down in T47D cells by infection with an shRNA vector. Two independent knockdown clones showing an approximately 70% decrease in β-catenin levels and a control LacZ shRNA were analyzed (Figure 4f). Treatment of both β-catenin knockdown clones and the control clone with Wnt1 CM led to a rapid increase in p-ERK1/2 levels to the same extent as seen in EGF-treated cells (Figure 4f). Taken together, these data demonstrate that, in human breast cancer cells, Wnt1 activates the ERK1/2 pathway in a WNT ligand- and DVL-dependent manner and this is independent of canonical signaling via β-catenin stabilization.

### Wnt1-induced ERK1/2 phosphorylation is EGFR-dependent

**Figure 5:**
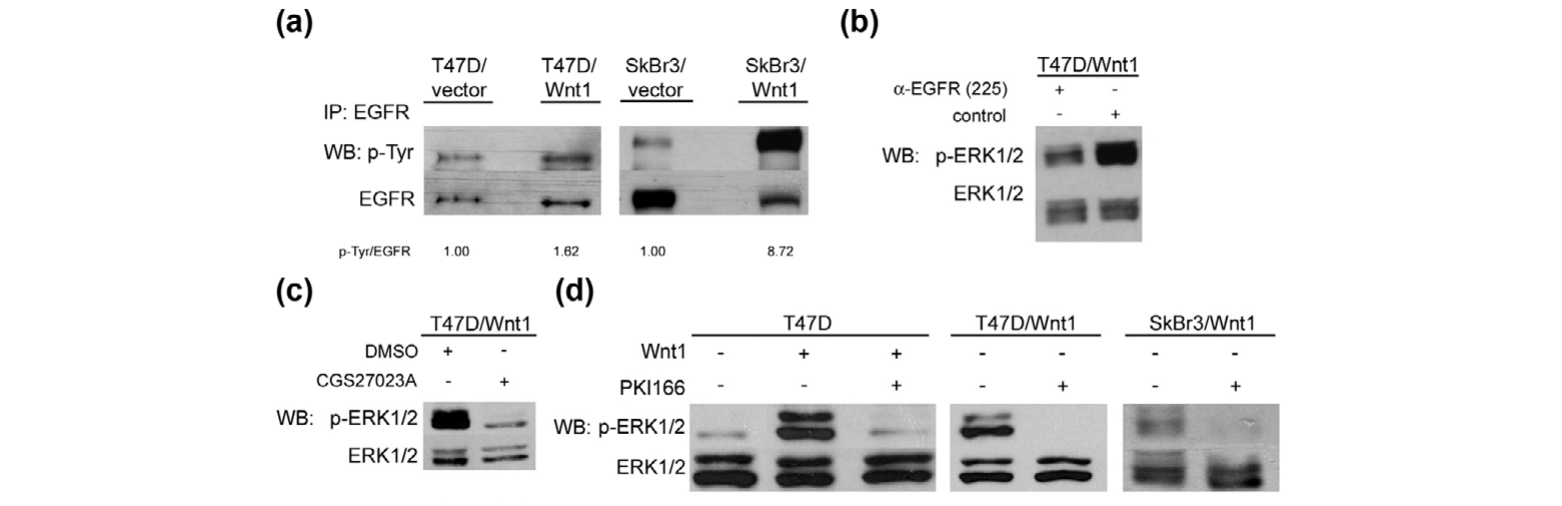
Wnt1-induced extracellular signal-regulated kinase 1/2 (ERK1/2) phosphorylation depends on epidermal growth factor receptor (EGFR) activity. **(a) **EGFR was immunoprecipitated from 2 mg of whole cell lysate from T47D/Wnt1, T47D/vector, SkBr3/Wnt1, and SkBr3 vector cells and analyzed by SDS-PAGE/immunoblotting for p-Tyr and EGFR. The p-Tyr signal corresponding to EGFR was quantified using the ImageQuant software. **(b) **T47D/Wnt1 cells were pre-treated for 1 hour with monoclonal antibody 225 containing conditioned medium (CM), and lysates were analyzed by SDS-PAGE/immunoblotting for p-ERK1/2 and ERK1/2. **(c) **T47D/Wnt1 cells were pre-treated for 1 hour with the metalloprotease inhibitor CGS27023A (50 μM) or dimethyl sulfoxide as control. Total lysates were analyzed by SDS-PAGE/immunoblotting for p-ERK1/2 and ERK1/2. **(d) **T47D cells were pre-treated for 1 hour with 2 μM PKI166 before treatment with Wnt1 CM or control CM. T47D/Wnt1 and SkBr3/Wnt1 cells were treated for 1 hour with 2 μM PKI166 prior to lysing the cells. Lysates were analyzed by SDS-PAGE/immunoblotting for p-ERK1/2 and ERK1/2. Tyr, tyrosine.

We next explored whether activation of EGFR is induced by Wnt1 and acts upstream of the observed ERK1/2 phosphorylation. Overall EGFR phospho-tyrosine levels are 1.6 fold and 8.7 fold elevated in T47D/Wnt1 and SkBr3/Wnt1 cells over the level in corresponding control transfected cells (Figure 5a). Treatment of T47D/Wnt1 cells with an EGFR-blocking antibody (225) that prevents ligands from binding the receptor causes a decrease in p-ERK1/2 to basal levels in the cells (Figure 5b). Shedding of EGF-like ligands from their membrane-bound form requires the activity of metalloproteases of the MMP and/or ADAM families. MMP or ADAM activity is required for the activation of the ERK1/2 pathway downstream of Wnt1 as the inhibitor of metalloprotease activity CGS27023A lowered Wnt1-induced ERK1/2 activity to basal levels (Figure 5c). Finally, the Wnt1-mediated increase in ERK1/2 activity was blocked by either pre-treatment of T47D cells or treatment of T47D/Wnt1 and SkBr3/Wnt1 cells with PKI166, an EGFR tyrosine kinase inhibitor (TKI) (Figure 5d) [37]. Taken together, these data suggest that Wnt transactivates EGFR via metalloprotease-dependent ligand release)[19].

### Wnt1-induced ERK phosphorylation requires Src kinase activity

**Figure 6:**
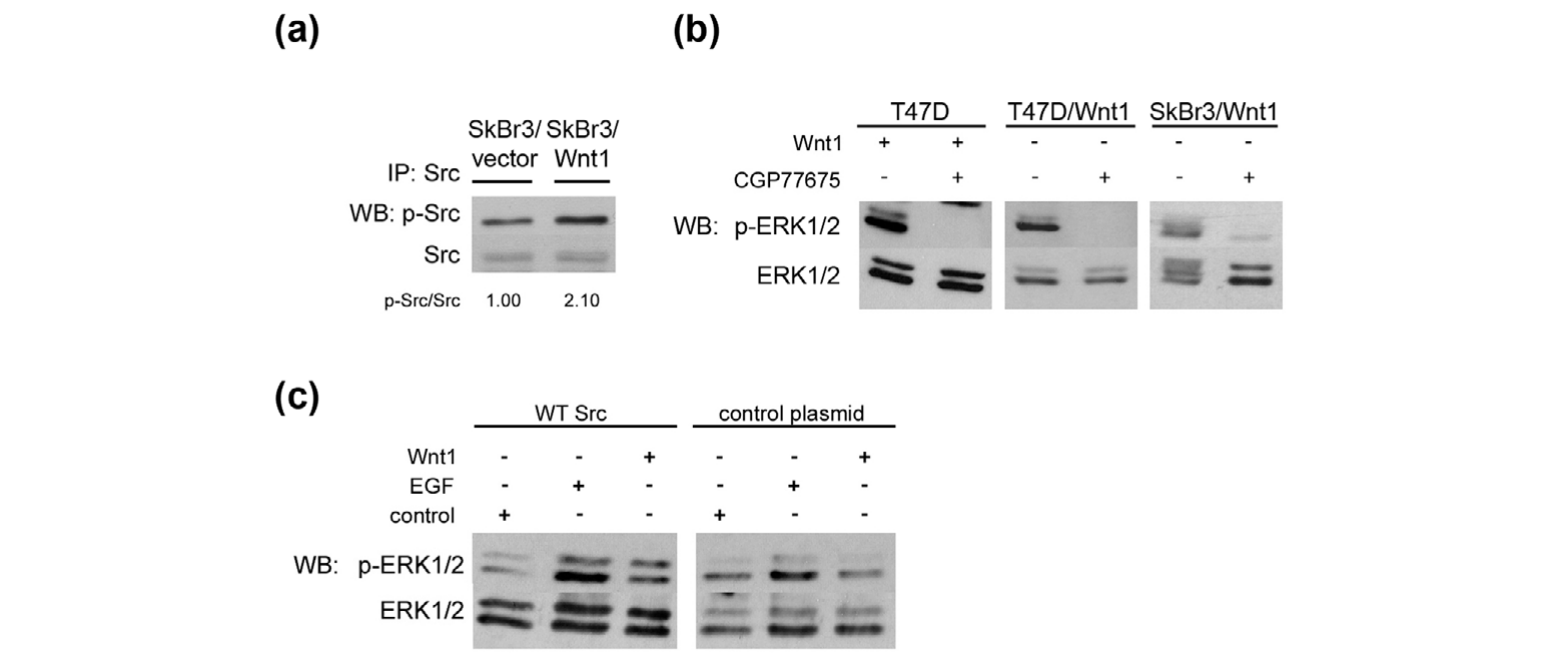
Src kinase is required for Wnt1-mediated extracellular signal-regulated kinase 1/2 (ERK1/2) activation. **(a) **c-Src was immunoprecipitated from lysates of Wnt1-expressing or vector control-expressing SkBr3 cells. The immunoprecipates were analyzed by SDS-PAGE/immunoblotting for p-Src and Src, and the signals were quantified versus control levels using the ImageQuant program. **(b) **T47D cells were pre-treated with the Src kinase inhibitor CGP77675 (2 μM) or dimethyl sulfoxide (DMSO) for 1 hour before treatment for 20 minutes with Wnt1 conditioned medium (CM). T47D/Wnt1 and SkBr3/Wnt1 cells were treated for 1 hour with CGP77675 or DMSO. Total lysates were analyzed by SDS-PAGE/immunoblotting for p-ERK1/2 and ERK1/2. **(c) **Src^-/- ^mouse embryonic fibroblasts transfected with a control plasmid or a wild type Src-expressing vector were treated with Wnt1 CM or control CM for 20 minutes. Total lysates were analyzed by SDS-PAGE/immunoblotting for p-ERK1/2 and ERK1/2. A representative result of three independent experiments is shown. EGF, epidermal growth factor; IP, immunoprecipitation; WB, Western blotting.

As FZD receptors are structurally related to GPCRs and members of the Src kinase family were reported to act in GPCR ligand-induced EGFR transactivation)[19], we explored the possibility that c-Src has a role in Wnt1-mediated EGFR transactivation. Initially, we tested whether Wnt1-expressing cells have elevated c-Src kinase activity by examining phosphorylation of the regulatory p-Tyr 416 in c-Src IPs. In SkBr3/Wnt1 cells, c-Src activity was increased two-fold over SkBr3/vector cells (Figure 6a). T47D cells have high levels of active c-Src, and no differences were observed between control and Wnt1-expressing cells (data not shown).

Next, we examined the effects of CGP77675, an Src kinase-selective TKI [38,39]. Treatment of T47D/Wnt1 and SKBR3/Wnt1 cells with CGP77675 lowered ERK1/2 activity. Moreover, induction of p-ERK1/2 mediated by Wnt1 CM was blocked by CGP77675 pre-treatment (Figure 6b). Since CGP77675 blocks the activity of multiple Src family members [38], we used MEFs from c-Src knockout mice that were transfected with a c-Src-expressing vector or a control vector to directly test the role of c-Src. Whereas EGF stimulated ERK1/2 activity in both cell lines (although slightly less in c-Src knockout fibroblasts) [39], Wnt1 treatment increased ERK1/2 activity in c-Src-transfected MEFs, but not in control MEFs (Figure 6c). Interference with intracellular Ca^2+ ^levels, PKC signaling, or Gα_i/o _signaling, each of which is known to impact on GPCR-induced EGFR transactivation, did not affect Wnt1-induced ERK1/2 phosphorylation (data not shown). These observations suggest that, as observed for many GPCR-activating ligands)[19], c-Src is also required for Wnt1-mediated EGFR transactivation.

### Wnt1 rescues breast cancer cells from growth arrest induced by anti-estrogen therapy

Ligand-mediated autocrine ERBB activation confers resistance to anti-cancer agents [22], including the ER antagonist 4-HT [40]. Based on the ability of Wnt1 to activate EGFR and ERK1/2 signaling in the ER^+ ^T47D and MCF-7 breast tumor cells (Figure 4a), we examined the effect of Wnt1 treatment on their response to 4-HT.

**Figure 7:**
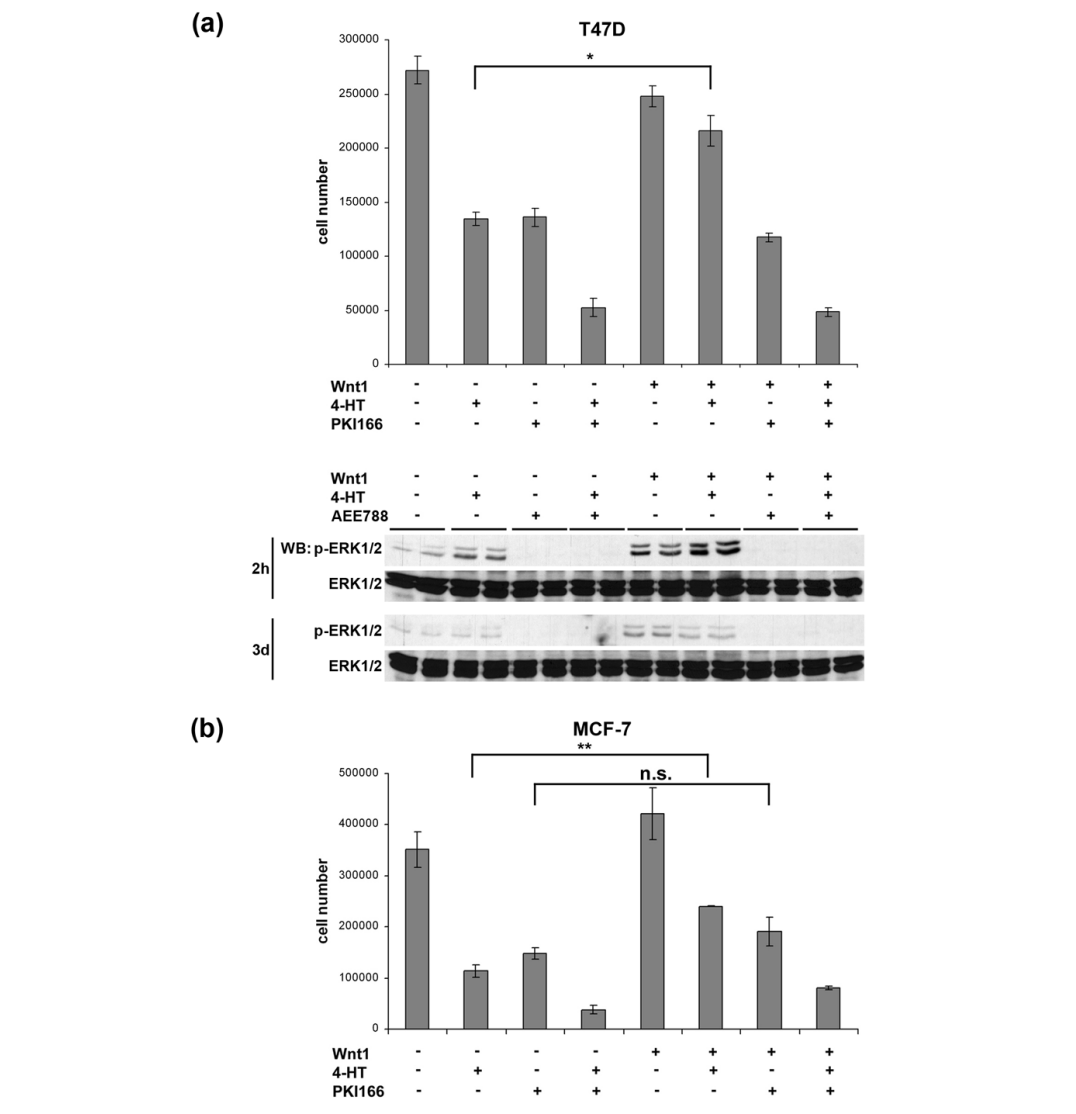
Wnt1 rescues breast tumor cells from the anti-proliferative effects of 4-hydroxytamoxifen (4-HT). T47D **(a) **or MCF-7 **(b) **cells were treated with 4-HT (5 μM), PKI166 (5 μM), or a combination of both drugs for 7 or 6 days, respectively, in the presence or absence of Wnt1 conditioned medium (CM) or control CM. For control experiments, the solvents ethanol and dimethyl sulfoxide, respectively, were added in corresponding concentrations. Cultures were re-fed with fresh medium and inhibitors after 3 days, before cell number (± standard error) was determined. For biochemical analysis, T47D cells were treated under corresponding conditions with 4-HT (5 μM), AEE788 (2 μM), or a combination of both in the absence or presence of Wnt1 CM or control CM for 2 hours or 3 days. Total lysates were analyzed by SDS-PAGE/immunoblotting for p-ERK1/2 and ERK1/2. *p < 0.05, **p < 0.005, unpaired Student *t *test. ERK1/2, extracellular signal-regulated kinase 1/2; n.s., difference not significant; WB, Western blotting.

Treatment of T47D and MCF-7 cells with 4-HT and PKI166 blocked proliferation by approximately 50% and 60%, respectively; the combination of inhibitors is essentially additive in both cell lines (Figure 7a,b). In T47D cells, Wnt1 treatment almost completely rescued the anti-proliferative effect of 4-HT (79% of control level, Figure 7a, upper panel). MCF-7 cells were also significantly rescued from the anti-proliferative activity of 4-HT by Wnt1 (32% of control level, Figure 7b). PKI166-treated T47D and MCF-7 cultures were both insensitive to Wnt1 addition, showing the dominance of EGFR blockade. Importantly, addition of PKI166 completely suppressed the ability of Wnt1 to overcome the anti-proliferative activity of 4-HT in both cell lines, showing the importance of autocrine EGFR activation in the Wnt1-induced rescue. In line with this, Western blot analysis reveals that the slight increase in p-ERK1/2 levels upon Wnt1 treatment observed after 2 hours of incubation (Figure 4e) is completely blocked using the more potent dual EGFR/ERBB2 kinase inhibitor AEE788 [41] while 4-HT treatment even enhances the activation of the ERK1/2 pathway slightly (Figure 7a, lower panel). After long-term treatment (3 days) with 4-HT in the presence of Wnt1, p-ERK1/2 levels are still elevated over basal levels, but ERK1/2 phosphorylation remains completely blocked by AEE788. These results imply that Wnt1 overcomes the anti-proliferative effect of anti-ER treatment in a manner that depends on EGFR activity.

## Discussion

De-regulation of WNT signaling is a well-established hallmark of certain types of human cancer, such as CRC and melanoma, in which a high percentage of mutations in the β-catenin destruction complex components *APC *and *AXIN *or in *β-catenin *itself have been described [42]. Although mutations of this type are rarely observed in breast cancer, we show here that many breast cancer cell lines have autocrine activity of WNT signaling and that blocking this pathway has multiple biological effects. In breast cancer, activation of the Wnt pathway is likely due to co-expression of WNT ligands and FZD receptors (T Schlange, unpublished observations) [7,12-14]. WNT ligands play different roles in cancer biology depending on the downstream pathways activated. Whereas canonical Wnt signaling is required for G_1 _cell cycle progression in CRC [43], the non-canonical ligand WNT5A negatively regulates proliferation [44,45] but promotes migration in various cancer types [46,47]. One potential mechanism contributing to pathway activity might be loss of negative modulators of WNT signaling [48], as decreased expression of sFRP1 is well documented in human breast cancer [10,11,29]. Furthermore, the loss of sFRP1 expression was recently shown to synergize with c-MYC-induced tumorigenesis [49]. Extending the analysis of Bafico and colleagues [21], we assayed the activation of WNT signaling by DVL phosphorylation, the most proximal read-out of FZD receptor activation, and found autocrine WNT activity in a panel of human breast cancer cells with diverse genetic alterations.

We show here that treatment of many breast tumor cell lines with sFRP1 has a consistently negative effect on their proliferation by affecting the canonical WNT pathway. In addition, ERK1/2 pathway activity is also decreased by sFRP1 treatment in the majority of the cancer cells, with SkBr3 cells being particularly sensitive. SkBr3 cells have high levels of ERBB activity. The fact that sFRP1 decreases p-ERK1/2 levels suggests that WNT-mediated ERBB transactivation has an important role in maintaining ERK1/2 signaling in these tumor cells. As SkBr3 cells have essentially no active β-catenin, sFRP1 effects on ERK1/2 activity might be the main cause for their decreased proliferation in response to sFRP1 treatment. A similar dependence on a non-canonical WNT signal was observed in β-catenin-deficient mesothelioma cells [50], in which siRNAs against WNT1 and DVL induced apoptosis in a JNK (c-jun N-terminal kinase)-dependent manner. This finding is particularly interesting given the inhibition of proliferation and induction of apoptosis we observe in response to the knockdown of all three DVL homologues in different breast cancer cell lines. Interfering with WNT signaling at the DVL level should block all autocrine activation [5]. sFRP1, on the other hand, most likely binds only a subset of WNT ligands [30,51], which might explain why sFRP1 treatment could not completely block β-catenin stabilization or WNT-induced ERK1/2 activity. In fact, compared with sFRP1 treatment, DVL knockdown elicited a stronger negative effect on ERK1/2 activity in the breast cancer cell lines. BT474 and MCF-7 cells are most resistant to both sFRP1 treatment and DVL knockdown when compared with the other cell lines analyzed. In the case of BT474, this is in line with relatively low levels of DVL phosphorylation, indicating that this cell line is mostly independent of autocrine WNT signaling. This shows that there is differential sensitivity of human breast cancer cells with different oncogenic pathways activated (for example, ERBB2 overexpression, estrogen dependence) to inhibition of autocrine WNT signaling.

Recently, blocking the FZD/DVL interaction using a small molecule targeting the PDZ domain of DVL was explored and shown to inhibit the proliferation of cancer cell lines derived from different types of cancer [52]. Our observations imply that targeting this interaction or the use of a 'ligand trap' like sFRP1 might be a valid approach to treat breast cancer by interfering with the canonical WNT pathway as well as the EGFR/ERK1/2 pathway. Inhibition of more than just one WNT ligand or FZD receptor may overcome the problem of functionally redundant expression of several family members when specific antibodies are used [53-57]. In summary, our observations on blocking autocrine WNT activity in human breast cancer cells suggest an important role for WNT-induced EGFR transactivation in the control of ERK1/2 signaling and of proliferation.

It is also noteworthy that there is differential phosphorylation of DVL isoforms in the panel of breast cancer cell lines. Perhaps DVL family members are not redundant and might be activated by different WNT/FZD complexes. Furthermore, DVL isoform levels vary substantially in different breast cancer cell lines. Therefore, it might be worth analyzing whether aspects of tumor biology like proliferation and migration are differentially regulated by these scaffolding proteins, potentially providing a paradigm for the differentiation of non-canonical versus canonical WNT signaling.

We show here that, in addition to activating the canonical Wnt/β-catenin pathway, Wnt1 transactivates EGFR and stimulates ERK1/2 activity in many human breast cancer cells. This Wnt1-mediated response is similar to EGFR transactivation induced by many GPCRs)[19]. In fact, various lines of evidence, including the GPCR-like heptahelical structure of the FZD receptor family and genetic data from *Drosophila*, suggest that these receptors have biological similarities [58-60]. Although we could not block Wnt1-induced ERK1/2 activation using pertussis toxin (PTX) to block Gα_i/o _proteins, this still leaves the possibility that PTX-insensitive Gα proteins mediate the effects of WNT/FZD signaling. Indeed, it was shown that Gα_q/11 _group proteins contribute to the activation of canonical WNT signaling [60,61].

Our results also show that c-Src has an important role in Wnt1-driven EGFR transactivation. Wnt1 was able to transactive EGFR in Src-expressing MEFs, but not in Src knockout MEFs. Furthermore, an Src kinase inhibitor abolished the effects of Wnt1 on ERK1/2 activation in human breast cancer cell lines and Src kinase activation was increased in SkBr3/Wnt1 cells. Src kinase has also been implicated in GPCR-mediated EGFR transactivation)[19]. Src kinase might act directly downstream of GPCRs and FZD receptors via its interaction with ADAMs and MMPs [62-65]. Association of Src kinases with these enzymes might regulate their proteolytic activity and subcellular localization [66], leading to an increase in ERBB ligand shedding and autocrine receptor activation. Since we observed that neither metalloprotease inhibitors nor an EGFR-blocking antibody completely blocked Wnt1-induced ERK1/2 activation, this might reflect a direct effect of Src kinase on EGFR activity due to its ability to phosphorylate the receptor at Tyr 845 [67]. The involvement of WNT-induced Src activity on EGFR activation is corroborated by our observation that the knockdown of DVL decreased the level of Tyr 845 phosphorylation in several breast cancer cell lines.

WNT signaling has previously been linked to the activation of Src and ERK1/2 in NIH3T3 cells and in osteoblast progenitors [68-70], and recently EGFR was shown to be involved in ERK1/2 activation downstream of purified Wnt3a [71]. However, these studies rely on overexpression or treatment with recombinant proteins and did not link the transactivation to autocrine signaling processes. It was recently shown that Wnt1 is induced by progesterone receptor (PgR) signaling in T47D breast cancer cells and that it is required for EGFR transactivation by a PgR agonist in an Src- and metalloprotease-dependent manner [72]. These results are interesting to consider in light of the data presented in this paper. It is possible that the rapid effects of steroid hormones leading to sustained proliferation or survival of breast tumor cells proceed by establishing an autocrine loop of EGFR activity that is linked, in part, to Wnt1 production. It will be important to see whether results from the T47D breast cancer model are clinically relevant in primary breast tumors, many of which overexpress Wnt1 [13].

EGFR activity is known to play a role in endocrine therapy resistance (for example, in MCF-7 cells) [73]. In fact, there are increased β-catenin levels and increased expression of WNT pathway target genes in these resistant cells [74], further implicating WNT pathway activity in endocrine resistance. Our data also show the potential importance of autocrine WNT signaling in response to anti-hormonal therapies. Wnt1 treatment of the ER^+ ^MCF-7 and T47D cells rescued them from the anti-proliferative action of 4-HT, and this was blocked by treatment with an EGFR TKI, showing the importance of autocrine EGFR signaling in the Wnt1 rescue.

## Conclusion

**Figure 8:**
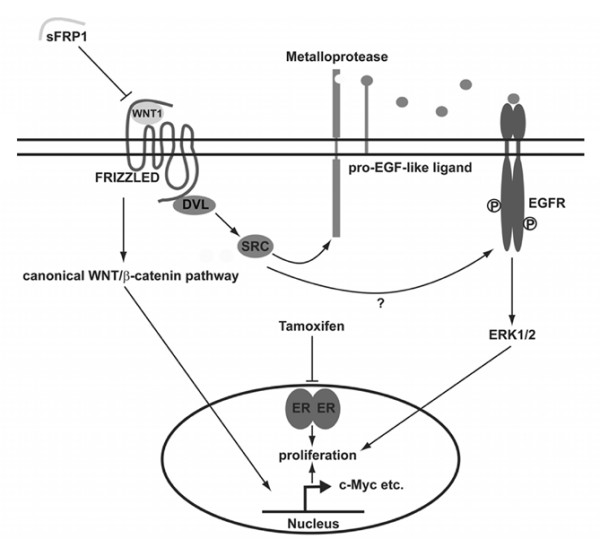
Schematic representation of wingless and integration site growth factor (WNT)-induced epidermal growth factor receptor (EGFR) transactivation. Our results show that Wnt1 induces a signaling cascade that links the activation of EGFR in a manner dependent on Dishevelled, SRC, metalloprotease, and EGF-like ligand to the sFRP1-sensitive activation of Frizzled receptors. The activation of EGFR, which may occur via phosphorylation of Y845, an SRC phosphorylation site, triggers activation of the extracellular signal-regulated kinase 1/2 (ERK1/2) pathway. Together with the canonical WNT/β-catenin pathway and its target genes, including *c-Myc*, the ERK1/2 pathway promotes proliferation and survival of breast cancer cells. Furthermore, activation of the ERK1/2 signaling by Wnt1 may contribute to the development of anti-estrogen resistance. DVL, Dishevelled; EGF, epidermal growth factor; ER, estrogen receptor; sFRP1, secreted Frizzled-related protein 1.

Our results support the concept that therapeutic interference with autocrine WNT signaling might be a useful strategy for targeting breast cancer. Furthermore, blocking the pathway at the level of WNT/FZD/DVL, in contrast to targeting the β-catenin/TCF complex [75], would not only impact on canonical signaling but also provide a novel interface for interfering with autocrine EGFR activity, an important target in breast cancer [22,32]. In Figure 8, we propose a model that incorporates the data presented in this paper.

## Abbreviations

4-HT = 4-hydroxytamoxifen; ADAM = A Disintegrin And Metalloprotease; CM = conditioned medium; CRC = colorectal cancer; DMEM = Dulbecco's modified Eagle's medium; DVL = Dishevelled; EGF = epidermal growth factor; EGFR = epidermal growth factor receptor; ER = estrogen receptor; ERK1/2 = extracellular signal-regulated kinase 1/2; FCS = fetal calf serum; FZD = Frizzled; Gα = heterotrimeric G protein subunit α; GFP = green fluorescent protein; GPCR = G protein-coupled receptor; HRP = horseradish peroxidase; IP = immunoprecipitation; MEF = mouse embryonic fibroblast; MMP = matrix metalloprotease; PARP = poly(ADP-ribose)polymerase; PgR = progesterone receptor; PTX = pertussis toxin; sFRP1 = secreted Frizzled-related protein 1; shRNA = short hairpin RNA; siRNA = short interfering RNA; TCF = T-cell factor; TKI = tyrosine kinase inhibitor; Tyr = tyrosine; WNT = wingless and integration site growth factor.

## Competing interests

The authors declare that they have no competing interests.

## Authors' contributions

TS designed and carried out the experiments, unless otherwise specified, and wrote the manuscript. YM analyzed the specificity of the act. β-catenin antibody. SL assisted TS in carrying out the experiments. AH purified sFRP1 protein. NEH participated in designing the experiments and in writing the manuscript. All authors read and approved the final manuscript.
